# Optimization of hybridization chain reaction for imaging single RNA molecules in *Drosophila* larvae

**DOI:** 10.1080/19336934.2024.2409968

**Published:** 2024-10-01

**Authors:** Julia Olivares-Abril, Jana Joha, Jeffrey Y. Lee, Ilan Davis

**Affiliations:** a Department of Biochemistry, University of Oxford, Oxford, UK; b School of Molecular Biosciences, College of Medical, Veterinary & Life Sciences, University of Glasgow, Glasgow, UK

**Keywords:** *Drosophila*, single-molecule imaging, smFISH, RNA visualization, RNA, fluorescence, HCR

## Abstract

*In situ* hybridization techniques are powerful methods for exploring gene expression in a wide range of biological contexts, providing spatial information that is most often lost in traditional biochemical techniques. However, many *in situ* hybridization methods are costly and time-inefficient, particularly for screening-based projects that follow on from single-cell RNA sequencing data, which rely on of tens of custom-synthetized probes against each specific RNA of interest. Here we provide an optimized pipeline for Hybridization Chain Reaction (HCR)-based RNA visualization, including an open-source code for optimized probe design. Our method achieves high specificity and sensitivity with the option of multiplexing using only five pairs of probes, which greatly lowers the cost and time of the experiment. These features of our HCR protocol are particularly useful and convenient for projects involving screening several genes at medium throughput, especially as the method include an amplification step, which makes the signal readily visible at low magnification imaging.

## Introduction

*In situ* hybridization (ISH) methods have been fundamental in widening our understanding of molecular mechanisms inside the cell, since they provide spatial information that is not available using biochemical methods requiring homogenization of tissues. The intracellular location of mRNA has been shown to play an important role in cell function, as localized protein translation provides a powerful mechanism for finely-tuned spatiotemporal control of protein synthesis in response to specific stimuli or changes in cell states [1–3]. Local mRNA translation is of particular interest in cells of the nervous system [4–7], whose role relies on their elongated morphology and ability to respond quickly to neuronal stimulation or signalling, which often occur at a substantial distance from the cell nucleus.

Since the invention of ISH techniques [8], the method has evolved from detection in formalin-fixed, paraffin-embedded tissues sections with radioactivity [9], to wholemount detection using histochemical staining [10], to detection by fluorescence (Fluorescence ISH, FISH) [11], culminating in the ability to resolve single-molecules of RNA (single molecule FISH, smFISH, [12]). Over the past few decades, the cost of oligonucleotide synthesis has dropped considerably, thus lowering the cost barrier for adopting the method. However, FISH methods remain expensive and complex, requiring synthesis of tens or even hundreds of DNA oligonucleotide probes and can suffer from background autofluorescence or poor penetration of probe into thick whole mounted samples [13]. These barriers to adoption have led to the development of highly specialized techniques, optimized for cost-effectiveness and efficacy in a variety of models [14].

**Figure 1.**
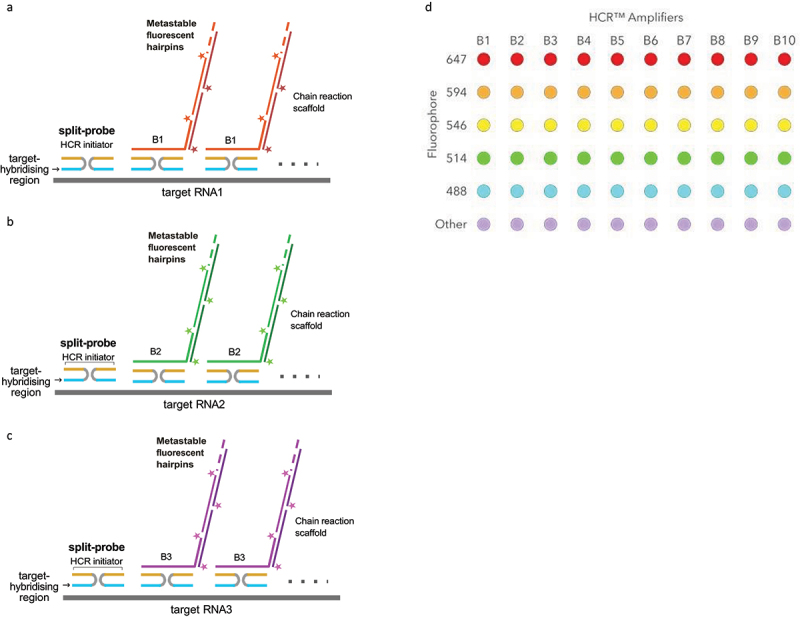
Mechanism by which HCR v3 binds target mRNA and generates fluorescent signal. (a, b, c) probes are designed to have three sections: a target hybridizing section (blue) that binds the mRNA of interest, a flexible connector (light grey) and one half of an HCR initiator sequence (yellow). The initiator is split between a pair of probes that bind the target mRNA sequentially to each other, so that when bound to the target mRNA they are close enough to reconstitute the full initiator sequence. This allows binding of the hairpin H1 (light red, light green, light purple), which is attached to a fluorophore. The hairpin input domain is exposed as a result, triggering binding of hairpin H2 (dark red, dark green, dark purple), also bound to a fluorophore. H1 and H2 polymerase and self-assemble into a fluorescent amplification polymer. For multiplexing, different initiator sequences (B1, B2, B3, etc.) are used, allowing for imaging of several RNAs of interest in the same sample using different colours (Figure 1a uses B1 initiator and a green fluorophore in probes against one RNA of interest, Figure 1b uses B2 initiator bound to a red fluorophore in probes against a different gene, and Figure 1c uses initiator sequence B3 and a far red fluorophore against a third RNA). (D)Shows the reagents available from molecular instruments for multiplexing (reproduced from: https://www.molecularinstruments.com/straightforward-multiplexing).

Here we present one such optimized hybridization chain reaction (HCR)-based method to detect individual mRNA molecules, suitable for imaging at low magnification and in specimens with significant autofluorescence, as it involves a signal amplification step. We demonstrate the utility of the method by applying it to ‘fillet-prepped’ *Drosophila melanogaster* larvae, in which internal organs are removed to expose the nervous system and body wall muscle, providing a system where the neuromuscular junction (NMJ) and brain can be imaged effectively in whole-mount in 3D. HCR was first described in 2004 [15], and has since evolved from an alternative to PCR to a FISH method that can be used in multiple parallel fluorescence channels on thicker and larger specimens with low powered objectives [16] thanks to its use of amplification to increase the fluorescent signal of each probe. Our optimization is based on the third generation of this application [17], which provides greater background suppression and higher specificity, and allows for multiplexed quantitative analysis. The main improvement of this iteration of HCR compared to its predecessors is the use of pairs of split initiator probes (Figure 1), where each of the split probes contains half of the target RNA sequence and half of an initiator sequence, instead of each probe carrying the full initiator sequence. As a result, when bound non-specifically, only half of the initiator sequence is present, which won’t generate amplification, whereas probes bound to the target RNA sequence will prompt the binding of a fluorophore-labelled hairpin H1. This binding exposes the input domain of H1, which binds the output domain of fluorophore-labelled hairpin H2, thus exposing its input domain and allowing formation of a tethered, self-assembling fluorescent amplification polymer. Polymerization of fluorescently tagged hairpins improves the signal-to-noise ratio, allowing deeper imaging in tissues and detection in lower power and numerical aperture objectives. Different initiator and hairpin sequences allow for multiplexing, and the amplification step greatly reduces the need for large numbers of probes against each single RNA molecule. This property reduces the cost of the method, allowing the screening of tens of different mRNAs with relatively modest budgets.

Our protocol adapts HCR v3 for its use on *Drosophila melanogaster*, particularly on larval nervous tissue, and includes a custom-made pipeline for optimized probe design, which allows for multiplexed, highly specific, bright imaging of RNA *in situ* with only five pairs of split probes per channel. We have compared the efficacy of the method with the gold standard single molecule FISH (smFISH) method and conclude that our optimization of the HCR method applied to *Drosophila* larvae, albeit producing slightly noisier images, results in a clear and accurate image while being cheaper and allowing for multiplexing and imaging at a lower magnifications.

## Materials and methods

### Drosophila genotypes and dissection method

For most experiments, Mdr65-Gal4;UAS-mcd8-GFP crawling L3 larvae expressing GFP in sub-perineural glia (kindly gifted by Rita Teodoro) were used. Oregon-R flies were used for the example illustrating multiplexing. Dissections were performed on Sylgard plates in Schneider’s media as described in previous publications [14]. Briefly, larvae are pinned on top and bottom and cut along the dorsal midline. Two pins are used on each side to flatten the body wall, and the gut is removed with forceps before fixation with para-formaldehyde (PFA).

### Probe design

**Figure 2.**
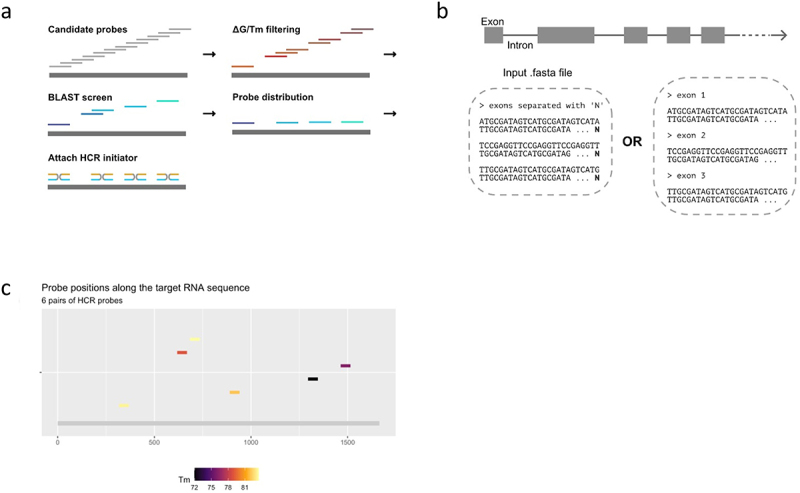
Custom probe-design pipeline, optimized for minimal off-target hits and maximum hybridization. (a) Steps of the probe design process: first, generation of all possible probes of the desired length so that they cover the entire length of the mRNA of interest. Next, filtering of these potential probes according to optimal thermodynamic properties and BLAST screen to remove off-targets. From those with appropriate thermodynamics and no off-targets, probes are selected so that they are distributed along the target sequence with no overlap. The software then splits the final candidates into two split probes, and attaches the flexible connector and the split initiator sequence for the hairpin selected out of the five available. (b) Different ways of indicating intron-exon junctions in the FASTA entry to avoid generation of probes spanning the junction region. (c) Output of the probe design software, showing final probe positions along the target RNA sequence, as well as the melting temperature of each of them.

We developed an open-source HCR probe designer software for *Drosophila* transcripts, which scans the target RNA sequence for optimal thermodynamic properties for hybridization while minimizing potential undesirable off-target hybridization (Figure 2a). Five HCR-B initiators (B1-B5) are available in the current version of the designer, and the user should select the initiator based on their multi-colour experimental design. Our probe designer software (https://github.com/jefflee1103/HCRv3_probe_design) and the user manual are available on an associated Zenodo publication, along with a custom *Drosophila melanogaster* transcriptome BLAST database [18]. Our designer is highly computationally efficient and is practical to use for probe design on a standard laptop computer (Intel Core i5, 8GB RAM, minimally).

The designer accepts an input DNA or RNA sequence as a FASTA format. For a successful HCR experiment, it is very important to consider at this stage whether you wish to detect all mRNA isoforms (constitutive) or select isoforms (isoform-specific) of a gene. Depending on the experimental design, the user can use transcript architecture to their advantage by choosing the sequence used to design probes (e.g. splicing isoforms, 3’UTR isoforms, retained introns). Additionally, it is important to consider exon-intron junctions carefully, as probes that span splice junctions will fail to detect pre-mRNAs (Figure 2b). This can be avoided by either (i) putting character ‘N’ between exons in case of a single FASTA entry; or (ii) having each exon as separate FASTA entries contained in a single FASTA file, which can be easily downloaded from the UCSC browser. The user guide in our Zenodo publication describes how to use the probe design software in further detail and recommends obtaining at least five pairs of split-probes for sufficient fluorescent signal (Figure 2C). The output oligos can be bought cheaply at minimum synthesis scales (~25 nmol) with standard desalting purifications in plate formats. We recommend pooling the probes at 100 µM as a stock concentration and keeping aliquots at 1 µM working concentration.

### HCR staining

Dissected larvae were fixed for 30 mins at room temperature in 4% PFA in PBSTx (0.3% triton X in PBS). They were rinsed three times in PBSTx, which was then used to permeabilize the tissue in two consecutive 20 min incubations at room temperature. Fixed and permeabilized larvae were transferred to 1.5 mL Eppendorf tubes filled with 5X SSCT (5X SSC, 0.1% tween) for 5 mins at room temperature. 5X SSCT was then replaced with wash solution (5×SSC, 30% formamide, 0.1% Tween) and the samples were incubated for 30 mins at 37°C, followed by two pre-hybridizations for 20 mins at 37°C in hybridization solution (5×SSC, 30% formamide, 10% Dextran sulphate, 0.1% Tween), and then hybridized overnight at 37°C in hybridization solution containing the probes (five pairs). To improve accuracy in the desired probe concentration of 10 nM, probes were first diluted to 1uM in TE. After the overnight incubation, probes were washed away with four 15 min washes in wash solution at 37°C. This was followed by two additional washes with SSCT at room temperature, for 5 mins each. Probes were then pre-amplified by incubating the samples in amplification solution (5×SSC +10% Dextran sulphate + 0.1% Tween) at room temperature for 30 mins. While this incubation took place, the hairpins were prepared for amplification. First, 0.5–2 mL of hairpin stocks were added to individual PCR tubes, aiming to have a final concentration of 60 nM of each hairpin in the hybridization step. Tubes were heated for 90 seconds at 95°C, and then immediately transferred to room temperature and cooled for 30 mins, protected from light. Hairpins were then pooled in amplification solution. The old amplification solution was removed and replaced by the one containing the hairpins, with which the samples were incubated overnight at 37°C, protected from the light. The next day, samples were rinsed three times in 5X SSCT, which was then used for two consecutive 30 min washes at room temperature. This was followed by a 2 h incubation with DAPI (0.02ug/L) and Dylight 405-conjugated HRP antibody (0.003ug/mL) for nuclear and neuronal membrane staining. Samples were rinsed twice with 5X SSC, followed by a 30 min wash with the same solution. 5X SSC was replaced with mounting medium (Slowfade diamond, Thermo Fisher scientific S36963) in which the samples were incubated for at least 10 min at room temperature before mounting for microscopy. All buffers can be aliquoted and kept at −20C.

### smFISH

smFISH was performed as described in [19]. In short, dissected larvae were fixed in PFA 4% PBSTx for 30 minutes at room temperature with gentle rocking, rinsed three times with PBSTx then washed twice in PBSTx for 20 minutes each. They were rinsed three times in wash buffer (2X SSC, 10% deionized formamide) and then incubated in the same solution for 30 minutes at 38°C. Wash buffer was replaced with hybridization buffer (2X SSC, 10% deionized formamide, 10% dextran sulphate and smFISH probes at a concentration of 250 nM), with which the samples were incubated at 38°C overnight. HRP-Dylight 405 was included at this step to label neurons. Probes were designed with the Stellaris Probe Designer (https://www.biosearchtech.com/support/tools/design-software/stellaris-probe-designer). Following the hybridization, samples were rinsed three times in wash buffer and then counterstained with wash buffer and DAPI (0.02ug/mL) for 30 minutes at 38°C. This was followed by three rinses with wash buffer and a longer 45 minutes wash at room temperature with gentle rocking. Wash buffer was then replaced with mounting media (Slowfade diamond, Thermo Fisher scientific S36963), in which samples were incubated for 30 minutes before mounting.

### Mounting and imaging

Specimen mounting was performed as previously described [14], placing the samples in a drop of mounting media placed between two strips of double-sided sticky tape to avoid damaging the sample between the slide and the coverslip and sealing it with transparent nail polish. Imaging was performed using either an Olympus/Evident SpinSR10 spinning disk confocal system with Prime BSI and Prime 95B sCMOS cameras and a 60X oil immersion (1.5 NA, UPLAPOHR60X) objective or another Olympus/Evident SpinSR10 spinning disk confocal system, fitted with a Prime BSI sCMOS camera and a 100X oil immersion (UPLXAPO100XO 1.45NA) objective. Olympus/Evident cellSens Dimension software were used for image acquisition.

### Image analysis

Images were stored on an OMERO server and data was visualized using Fiji/ImageJ. Quantification and image analysis was performed using a custom pipeline (https://github.com/jefflee1103/Image-analysis/tree/main/BigFISH) based on BigFish [20].

### Dnase and RNAse treatment

Pre-treatments with DNAse and RNAse took place immediately after permeabilization. After fixing and permeabilizing by washing three times in 0.3% PBSTx, samples were washed in PBS Tween (0.1%) for five minutes. For DNAse pre-treatment, samples were then washed in DNAse buffer (TURBO DNAse buffer, AM2238) for ten minutes before an overnight incubation at 37°C in TURBO buffer and 1 mm magnesium chloride with DNAse (TURBO DNAse, AM2238, 0.02 U/mL). For RNAse pre-treatment, samples were directly incubated overnight at 37°C in PBS Tween 1% and 1 mm magnesium chloride with RNAse T1 (EN0541, 20 U/mL) and RNAse III (AM2290, 0.02 U/mL). The next day, HCR protocol was continued as described previously, starting from the first wash with 5X SSCT.

## Results

**Figure 3.**
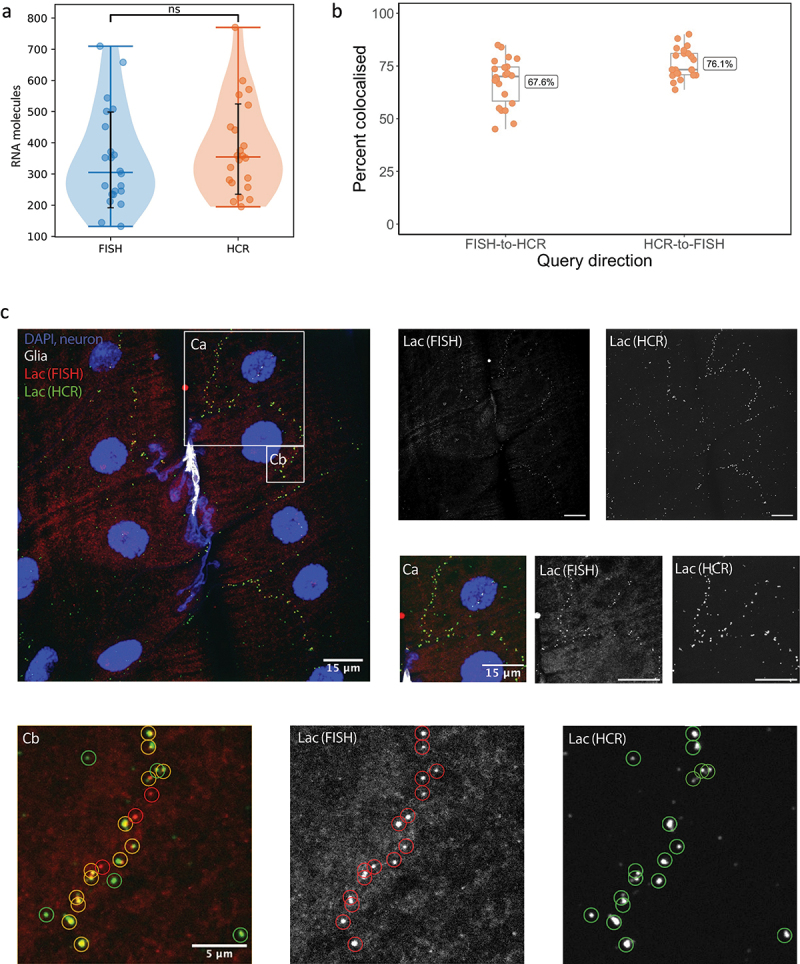
Comparison of our optimized HCR method and smFISH in *Drosophila* melanogaster neuromuscular junction. (A) quantification of spots detected by each technique in neuromuscular junction samples after performing both smFISH and HCR against lachesin (lac) mRNA in fillet-prepped larvae (*n*=22). (B) Colocalization analysis of smFISH and HCR, showing the percentage of spots detected with one technique that are within a radius of <750nm of a spot detected with the other technique. Analysis was performed twice, once with smFISH as a starting point (67.6%) and once with HCR as a starting point (76.1%). (C) larval neuromuscular junction with cell nuclei (DAPI) and neurons (HRP-Dylight 405) presented in blue, glial cells in white (mdr65>GFP), smFISH probes against lac mRNA in red and HCR probes against the same gene in green. (ca) highlights a region of the cell where the mRNA is most present. (cb) circles the spots detected with each technique (smFISH in red, HCR in green, yellow circles for colocalized spots).

Starting from well-established HCR protocols [21], we compared our optimized protocol using only five pairs of probes with a state-of-the-art smFISH technique previously described by our lab [14] by including both HCR and FISH probes against *lachesin* (*lac*) mRNA in the same sample. Both techniques produced very similar results (Figure 3), although we found that HCR produced an average of approx. 10% more spots per sample (Figure 3a, average of 380 spots detected using HCR, 345 using smFISH), showing that HCR suffers from more non-specific probe binding than smFISH. We compared more precisely the non-specific signal in the two methods samples by studying the colocalization of the HCR and smFISH puncti (Figure 3b). We found a higher percentage of colocalization (76.1%) when calculating the percentage of smFISH puncti that are colocalized with an HCR spot, compared with the HCR puncti colocalized with smFISH spots (67.6%). Most of the difference can be attributed to floating self-assembled hybridized initiators, which are not bound to RNA molecules and can be seen less bright and mobile under the microscope. However, both HCR and smFISH produced a very similar pattern of spots (Figure 3c, ca, cb), giving us confidence in the reliability of our method even with the reduced number of probes used against each gene of interest. This is particularly true for our purpose of a wider-scope screen, especially given how variable mRNA numbers for the same gene and conditions are [22]. Another difference to note is that smFISH has lower variation in the intensity of individual molecules than HCR, thus enabling a more precise quantitation of clusters of multiple mRNA molecules, such as those at transcription sites. However, individual molecules can only be reliably detected using smFISH with the highest numerical aperture high magnification objectives.

Nevertheless, we tend to try to optimize all FISH experiments to minimize background and non-specific signal. When troubleshooting, we recommend increasing washing times in the steps after hybridization, and tightly controlling the intensity threshold at the quantification step during image analysis.

**Figure 4.**
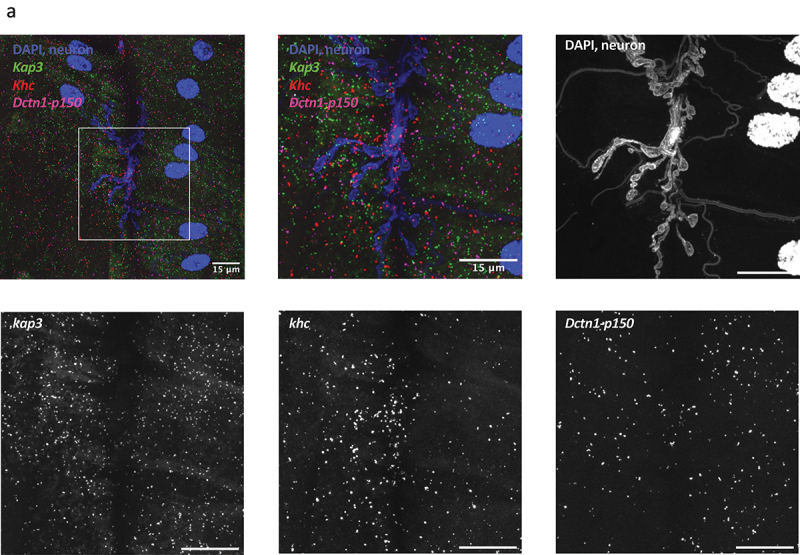
Detection of multiple mRNAs in the same sample with HCR. Cell nuclei (DAPI) and neurons (HRP-Dylight 405) are shown in blue, HCR probes against Kap3 in green, against khc in red, and against Dctn1-p150 in magenta. A section of the neuron is presented zoomed in for ease of visualization.

To reduce sample preparation time and reagent used, it was important that the technique allowed for multiplexing [23,24]. We tested HCR probes with initiator sequences B1, B2 and B3 (Figure 1) against three of our RNAs of interest, *dctn1-p150*, *kap3* and *khc*. The resulting images show markedly different distributions of each RNA, proving that our HCR method optimization allows for imaging of multiple genes in the same sample (Figure 4). Due to fluorescent spectral overlap, we were limited to using up to five colours at one time, each associated to one hairpin (B1-B5) and one RNA of interest. However, it is possible to increase the number of RNAs detected in any given sample using orthometric multicolour encoded hybridization chain reaction amplifier (multi-HCR), which uses a genetic barcode system to allow for one RNA to bind combinations of fluorophores [25].

**Figure 5.**
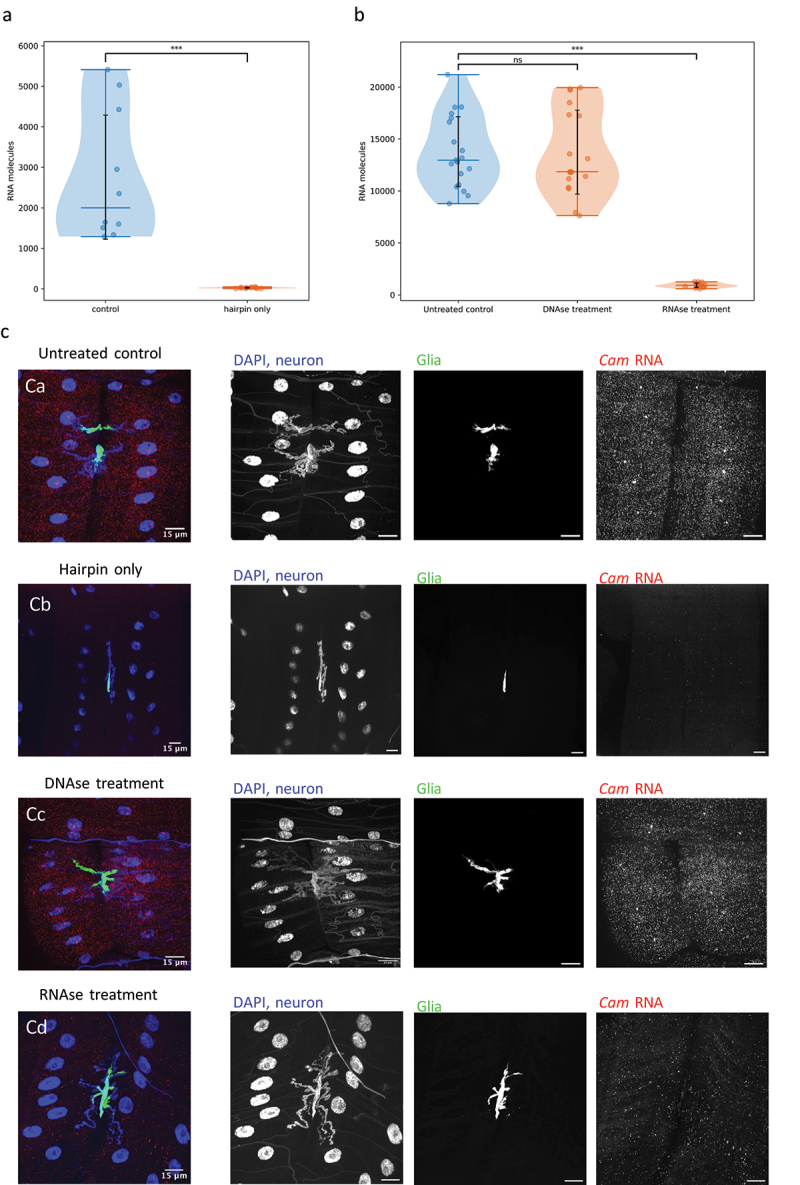
Analysis of controls. (a) Quantification of spots detected by HCR against Cam mRNA (ubiquitously expressed) in control samples where the protocol was performed normally (*n*=10) and in samples where the hairpins were added, but no probes against the mRNA were included (*n*=11). (b) Quantification of spots detected by HCR against Cam mRNA in control samples (*n*=19) and in samples treated with DNAse (*n*=16) and RNAse (*n*=12). (c) Images of *Drosophila* larval neuromuscular junction images showing cell nuclei (DAPI) and neurons (HRP-Dylight 405) in blue, glia (Mdr65>GFP) in green, and HCR probes against Cam mRNA in red. (ca) is an untreated control, where the HCR protocol was performed normally, (cb) is from a sample incubated with hairpins but without HCR probes, (cc) is from a sample treated with DNAse, and (cd) from a sample treated with RNAse.

To control the reliability of our protocol, we tested for background signal generated by non-specific amplification. For this purpose, we performed the experiment without including probes against any RNAs, which showed that the hairpins alone produce a negligible amount of signal (Figure 5A, Cb). We also tested the protocol on samples pre-treated with DNAse and RNAse to ensure the spots accurately represent RNA molecules, using probes against *Cam* RNA, a ubiquitously expressed gene. The DNAse-treated control showed a reduction of intensity of the nuclear staining with DAPI, but a negligible reduction in number of spots (Figure 5B, Cc). In contrast, RNAse treatment resulted in a marked decrease in number of spots (Figure 5B, Cd). We tested increasing concentrations of RNAse and detergent, and the HCR signal decreased when compared with lower concentrations (data not shown), but nevertheless, some signal remained. We interpret these results as indicating that the remaining signal is due to RNAse failing to fully penetrate deep into the thick NMJ sample. From these controls, we conclude that the vast majority of the signal we detect by HCR is a result of binding of the probe to the RNA of interest and of the fluorescent hairpin to the probe.

## Discussion

Here, we describe our optimization of HCR for quantification of RNA molecules *in situ* in the *Drosophila melanogaster* larval NMJ preparations. Our method is based on HCRv3, which uses a split initiator probe to minimize non-specific binding. Our optimization produces a clear, quantifiable image of the intracellular localization of up to five RNAs of interest at once. We also provide an open-source software tool that simplifies and speeds up the probe design process and generates high affinity, high specificity probes capable of producing high quality staining with as little as five pairs of probes. Use of additional probe pairs will produce a better signal-to noise ratio and can be used for experiments that require more fidelity [21], however we found that five pairs are sufficient to obtain a good quantitative readout of RNA localization. We find that when using FISH in higher throughput applications using lower magnification imaging, HCR is superior to smFISH, despite its higher non-specific signal.

In the future, we expect that editing the probe design pipeline to introduce multi-HCR [25] will allow for detection of up to 31 RNAs of interest in one sample by combining the five available different hairpin colours without having to manually conjugate probes with fluorophores. This will further speed up the screening process and lower the cost by reducing time and reagents consumed per gene studied. Having a technique that allows for efficient RNA localization screening provides a unique opportunity to add biological context to other quantitative RNA analysis techniques, such as single-cell RNA sequencing, as it can act as a direct read-out of these experiments.

However, it is important to note that this optimized method, particularly with a reduced amount of probes, does sacrifice some accuracy in the benefit of a low cost per experiment when compared to other techniques. In contrast to HCR, smFISH produces less noise and has lower variation in the intensity of individual molecules so enables a more precise quantitation of clusters of multiple mRNA molecules, such as those at transcription sites. However, individual molecules can only be reliably detected using smFISH with the highest numerical aperture high magnification objectives. Therefore, HCR is more indicated for screening experiments rather than detailed insights into one specific gene, for which a more expensive but more accurate technique such as smFISH would be more advantageous. However, the simple protocol, low cost, high-intensity signal and possibility to multiplex of HCR make it an ideal candidate for the purpose of screening subcellular RNA location.

In conclusion, the optimized method presented here provides a useful tool for visualizing RNA *in situ* in *Drosophila* in an inexpensive and more efficient way than other FISH methods. Our protocol is particularly useful for screening of large numbers of genes, and its ease and low cost provide an opportunity to study sub-cellular localization at a larger scale than was previously possible.

## Data Availability

The data supporting the findings of this study is available in the article.
